# Individual and Synergistic Anti-Coronavirus Activities of SOCS1/3 Antagonist and Interferon α1 Peptides

**DOI:** 10.3389/fimmu.2022.902956

**Published:** 2022-06-21

**Authors:** Chulbul M. Ahmed, Tristan R. Grams, David C. Bloom, Howard M. Johnson, Alfred S. Lewin

**Affiliations:** ^1^ Department of Molecular Genetics and Microbiology, University of Florida, Gainesville, FL, United States; ^2^ Department of Microbiology and Cell Science, University of Florida, Gainesville, FL, United States

**Keywords:** antivirals, SARSCoV-2, SOCS1/3 antagonist, interferon, beta-coronavirus

## Abstract

Suppressors of Cytokine Signaling (SOCS) are intracellular proteins that negatively regulate the induction of cytokines. Amongst these, SOCS1 and SOCS3 are particularly involved in inhibition of various interferons. Several viruses have hijacked this regulatory pathway: by inducing SOCS1and 3 early in infection, they suppress the host immune response. Within the cell, SOCS1/3 binds and inhibits tyrosine kinases, such as JAK2 and TYK2. We have developed a cell penetrating peptide from the activation loop of the tyrosine kinase, JAK2 (residues 1001-1013), denoted as pJAK2 that acts as a decoy and suppresses SOCS1 and 3 activity. This peptide thereby protects against several viruses in cell culture and mouse models. Herein, we show that treatment with pJAK2 inhibited the replication and release of the beta coronavirus HuCoV-OC43 and reduced production of the viral RNA, as measured by RT-qPCR, Western blot and by immunohistochemistry. We confirmed induction of SOCS1 and 3 in rhabdomyosarcoma (RD) cells, and this induction was suppressed by pJAK2 peptide. A peptide derived from the C-terminus of IFNα (IFNα-C) also inhibited replication of OC43. Furthermore, IFNα-C plus pJAK2 provided more potent inhibition than either peptide alone. To extend this study to a pandemic beta-coronavirus, we determined that treatment of cells with pJAK2 inhibited replication and release of SARS-CoV-2 in Calu-3 cells. We propose that these peptides offer a new approach to therapy against the rapidly evolving strains of beta-coronaviruses.

## Introduction

Beta-coronaviruses are enveloped viruses with a large positive-stranded RNA genome that infect humans as well as other mammals (1). Three beta-coronaviruses have caused human pandemics, SARS (of the 2002/2003 China pandemic), SARS-MERS-CoV (of the 2012 Middle East pandemic), and SARS-CoV-2, the coronavirus that is responsible for the ongoing global pandemic [reviewed in (2, 3)]. The existence of these extremely pathogenic beta-corona viruses with high rate of morbidity and mortality raises the possibility of emergence of newer strains that we must prepare for. Seasonal beta-coronaviruses, such as human coronavirus OC43 (HuCoV-OC43) can cause respiratory infections with varying degrees of morbidity (4, 5). Even so, it has been hypothesized that immunity against the seasonal common cold coronavirus OC43 mitigates against the highly contagious and much more virulent SARS-CoV-2 virus that is responsible for COVID-19 (6, 7). Antibody screening and epitope mapping indicate that a conserved region of spike protein 2 (SP2) near the fusion peptide is partly responsible for the cross protection between OC43 and SARS-CoV-2 (8) and vaccination for SARS-CoV-2 elevates neutralizing antibodies against seasonal human coronaviruses (9). These findings indicate that studying seasonal coronaviruses such as OC43 is potentially valuable in the search for a universal therapeutic for the various variants of SARS-CoV-2 as well as their less virulent relatives.

There are several antiviral drugs currently used to treat COVID19 infections. The first, of modest effect, is the antiviral drug remdesivir, a ribonucleoside analogue that inhibits virus nonstructured protein RNA-dependent RNA polymerase, which is likely to have side effects in causing mutations in the host (10). Because hydroxychloroquine interferes with endosome acidification and inhibits replication of SARS-CoV, it was believed that this drug might be an effective treatment for SARS-CoV-2 but SARS-CoV-2 is activated by TMPRSS2 rather than Cathepsin L (11), and this drug is ineffective against COVID-19 (12). Recently, molnupiravir, a ribonucleoside analog that causes errors in SARS-CoV-2 RNA synthesis, has been approved by the FDA for emergency treatment of COVID-19 (13). Another drug, paxlovid, a SARS-CoV-2 3CL protease inhibitor, has also been recently approved by the FDA under Emergency Use Authorization for treatment of mild to moderate COVID19 disease (13). These latter two drugs are currently the leading candidates for a therapeutic approach to COVID19. These drugs were not designed specifically for COVID19. Molnupiravir, for example, was designed to treat other RNA viruses such as influenza virus, which is a negative stranded segmented RNA virus (14). The known and potential risk to germ cells, along with generic gastrointestinal effects, suggests that it is worth pursuing the development of additional antivirals.

The interferon (IFN) system is the primary innate and adaptive immune response to viruses, including SARS-CoV-2 (15). There have been demonstrations that IFN is effective against SARS-CoV-2 in cell cultures (16, 17). The process of activation of cells by cytokines, such as IFNs, in response to viral infections also activates an inducible cytokine regulatory system called suppressors of cytokine signaling (SOCS) (18). Among these, SOCS1 and SOCS3 are particularly important in neutralizing interferons (19). SOCS proteins represent an important group of checkpoint inhibitors that act in concert with the program death (PD-1) protein and its ligand (PD1-L) and the B7 related CTLA-4 protein (20–22). Unlike the PD-1/PD-1L and CTLA4 (23), the importance of SOCS as checkpoint inhibitors is not widely appreciated.

We have shown that SOCS1 and SOCS3 (SOCS1/3) function as virus induced intrinsic virulence factors for influenza virus, EMC virus, herpes simplex virus 1 (HSV-1), vaccinia virus and influenza A infections (2, 24–28). Other viruses such as pathogenic pig enteric coronavirus and coronavirus induced severe acute respiratory syndrome (SARS) spike protein also induce SOCS virus intrinsic virulence factors (29). SOCS1/3 exert their viral virulence effect *via* inhibition of various interferons (30, 31). Specifically, the SOCS proteins bind to the activation loop of receptor-associated tyrosine kinases JAK2 and TYK2 through the SOCS kinase inhibitory region (KIR) inhibiting the activation of STAT transcription factors by the kinases. Activated STATs are required for IFN function. We developed a small peptide antagonist of SOCS1/3 that blocks SOCS1/3 inhibitory activity and prevents virus pathogenesis. The antagonist, pJAK2 (1001–1013), is comprised of the JAK2 activation loop, phosphorylated at tyrosine 1007 with a palmitate (lipo) for cell penetration. The remarkable thing about the SOCS1/3 antagonist is that it serves as a broad, simple tool of perhaps most pathogenic viruses to avoid innate host IFN defense (2, 27). In addition, we have generated data over at least two decades that has resulted in a non-canonical model of IFN signaling (32, 33). The model has permitted the development of small peptide mimetics of type I and type II IFN that possess potent antiviral activity but lack the toxicity associated with intact IFNs (25, 34). One of the mimetic peptides comprises the C-terminus of human IFNα with a lipid moiety to facilitate cell penetration, Lipo-IFNα1 (152–189), showed potent antiviral activity for encephalomyocarditis virus (25). Human IFNα and IFNβ and ovine IFNτ C-terminus mimetics showed potent antiviral activity against vaccinia virus and encephalomyocarditis (EMC) virus in cell culture and in infected mice (25).

In this study, we demonstrate that the SOCS1/3 antagonist peptide (pJAK2) inhibits cell toxicity and viral release of HuCoV-OC43 in cell culture. We also show that infection of cells with this virus induces SOCS1 and SOCS3, and that pJAK2 eliminates this induction. Similarly, the interferon mimetic peptide (IFNα−C) blocked viral replication and reduced cell toxicity by HuCoV-OC43. Furthermore, the combination of these two treatments leads to more pronounced inhibition of viral toxicity and replication than treatment with pJAK2 or IFNα-C alone. Finally, to determine if this approach might be effective against the agent of COVID-19, we show that pJAK2 peptide blocks the replication and release of SARS-CoV-2. Unlike the current drugs, these peptides are components of endogenously produced proteins. The findings suggest a novel approach to treating viral disease that tap into well-known immune modulators.

## Materials and Methods

### Cell Culture and Antiviral Assays

Human muscle rhabdomyosarcoma cells, RD (ATCC, CCL136) were grown in DMEM with 10% Fetal Bovine Serum (FBS, Thermo Fisher) and 1% penicillin streptomycin solution (Pen-Strep) in a 37°C humidified incubator with 5% CO_2_. Monkey kidney epithelial Vero-E6 cells (ATCC, CRL1586) were grown in MEM containing 10% FBS and 1% Penn-Strep. Human lung adenocarcinoma epithelial Calu-3 cells (ATCC, HTB-55) were grown in EMEM with 10% FBS and 1% Penn-Strep. Human beta-coronavirus HuCoV-OC43 (a gift from Dr. John Lednicky, University of Florida) was handled in a BSL-2 facility. Infection with HuCoV-OC43 in RD cells was carried out at MOI of 0.1 for 1 hr, followed by washing and further growth in 2% FBS containing media, called as low serum media henceforth. In experiments where cell viability was measured, at the end of incubation, CellTiter reagent (Promega, Madison, WI) was added and incubated further at 37° C for 1 hr before reading the color development in a plate reader at 495nm. When the effect of peptides was investigated, cells were first pre-incubated with the desired concentrations of the peptides for 1 hr and infected with HuCoV-OC43 for 1 hr. Cells were washed with phosphate buffered saline (PBS), and the same concentrations of peptides were added and incubated in 2% FBS containing media (low serum media) for 48 hours, to avoid overcrowding of cells, as described in (35). Cell extracts and supernatants were harvested. For measurement of viral titers on Vero-E6 cells, aliquots of serial dilutions of cell supernatants were added for 1 hr, washed and incubated for an additional 72 hr. Cells were stained with crystal violet and the number of plaques was counted.

SARS-CoV-2 strain UF-1 (GenBank accession number MT295464.1) was isolated from a COVID-19 patient at the University of Florida, and handled in a Biosafety Level 3 (BSL3) laboratory under a protocol approved by the University of Florida Institutional Biosafety Committee. The virus was propagated on Calu-3 cells and the dilutions of supernatants were seeded on Vero-E6 cells for titration.

### Peptide Synthesis

SOCS1/3 antagonist peptide (pJAK2), and its inactive control peptide (JAK2A) were synthesized by GenScript (Piscataway, NJ). Poly-arginine (R9) on the N-terminus was included in the synthesis to make the peptide cell permeable. The sequence of pJAK2 is (R9)LPQDKEpYYKVKEP, where pY stands for phosphotyrosine that was critical for its biological activity. In the control peptide the tyrosines were replaced by alanines, and had the sequence, (R9)LPQDKEAAKVKEP. These peptides were dissolved in PBS before use. The peptide IFNα−C consists of the C-terminus of human IFNα1 from the residues 152 to 189. A palmitoyl-lysine was attached to the N-terminus of this peptide as a last step in synthesis to allow it to cross the plasma membrane. IFNα−C peptide was synthesized by GenScript (Piscataway, NJ). It was dissolved in dimethylsulfoxide (DMSO) at 10 mg/ml before use. Further dilutions were carried out in PBS. Appropriate amount of DMSO was used as vehicle control.

### RNA Extraction and qPCR Analysis

For HuCoV-OC43 studies, RD cell culture extracts or supernatants were dissolved in Trizol reagent (Invitrogen) and homogenized using a hand-held homogenizer (Kimble). Direct-zol RNA kit from Zymogen Research (Irvine, CA) was used for RNA extraction. One microgram of RNA was used to synthesize cDNA using iScript kit from Bio-Rad (Hercules, CA) following the manufacturer’s instructions. Quantitative PCR was carried out with sSoAdvanced PCR kit from Bio-Rad using the conditions described in (36). PCR primers used are listed in **Table 1** and were synthesized by Eurofins. Relative target gene expression was normalized to β-actin as an internal control using ^ΔΔ^Ct method (37).

**Table 1:** Nucleotide sequence of the primers used in qPCR experiments.

OC43RdR-F:	5’-GAGTGTAGATGCCCGTCTCG-3’
OC43RdR-R:	5’-TGTGGCACACGACTACCTTC-3’
SOCS1-F:	GGAACTGCTTTTTCGCCCTTA
SOCS1-R:	AGCAGCTCGAAGAGGCAGTC
SOCS3-F:	GACCAGCGCCACTTCTTCA
SOCS3-R:	CTGGATGCGCAGGTTCTTG
SARS-CoV-2N-F:	5’ – GGGAGCAGAGGCGGCAGTCAAG – 3’
SARS-CoV-2N-R:	5’ – CATCACCGCCATTGCCAGCCATTC – 3’
SARS-CoV-2N-Probe:	5’ FAM -CCTCATCACGTAGTCGCAACAGTTC- BHQ1-3’

Abbrev: RdR, RNA dependent RNA polymerase from HuCoV-OC43; CoV-2N, CoV-2 Nucleocapsid protein.

For analysis of transcripts from SARS-CoV-2 infected Calu-3 cell supernatants were harvested 24 hrs after infection. Briefly, 100 μL of viral supernatant was used for total RNA extraction using the QIAamp viral RNA mini kit (Qiagen). Real-time quantitative RT-PCR was used to quantify SARS-CoV-2 replication using QuantiNova Probe RT-PCR kit (Qiagen) with a StepOne Plus Real-Time PCR system (Applied Biosystems). The number of copies of the RdRP/hel were calculated using an RdRP cDNA standard curve. Primers and probes were used against the RNA-dependent RNA polymerase and helicase gene region of SARS-CoV-2 as listed in **Table 1**. Cycling conditions were 20 min at 50°C for reverse transcription step, followed by 2 minutes at 95°C for Taq polymerase activation step, then 44 cycles of 15 sec. at 95°C of denaturing, 30 sec. at 57°C for annealing, and 20 sec. at 68°C for extension, as described previously (1).

### Immunohistochemistry

RD cells were grown in eight-well chambered slides and grown overnight to a 70% confluency. We pre-treated with the peptides indicated for 1 hr followed by infection with HuCoV-OC43 (at MOI of 0.1) for 1 hr. The cells were washed with media without serum and suspended in medium containing 2% FBS with the same amount of peptides and incubated further for 48 hrs. We then fixed cells with 4% paraformaldehyde for 30 min, followed by permeabilization with PBS containing 1% Triton X-100 for 30 min at room temperature. Cells were blocked in 10% normal goat serum in PBS with 0.5% Triton X-100 for 30 min at room temperature, followed by two washing in PBS with 0.2% Tween-20 (wash buffer), for 10 min each. Mouse monoclonal antibody to HuCoV-OC43 nucleoprotein (Sigma, cat no. MAB9013) was added at a dilution of 1:500 in PBS with 0.2% Tween-20 for 2 hrs at room temperature. Cells were washed 4 times with wash buffer. We incubated with Texas red conjugated secondary anti-mouse antibody (for pJAK2 treatment) and DAPI for 30 min. Antibody treatment was followed by four washings and the addition of mounting media (Southern Biotechnology), and covering with a cover slip. When cells were treated with IFNα−C, we used AlexFluor 488 conjugated anti-mouse antibody (Invitrogen) and DAPI for staining. We imaged cells using a Keyence BZ-X700 fluorescence microscope.

### Western Blot Analysis

RD cells were treated with 30 μM each of pJAK2, IFNα−C or the control peptide for 1 hr followed by infection with HuCoV-OC43 at an MOI of 0.1 for 1 hr. Cells were washed and suspended in low serum medium containing the same amount of peptides and incubated for 48 hr. Cells were washed and suspended in RIPA buffer with protease inhibitors (Santa Cruz Biotechnology) and frozen. Equal amounts of protein were separated by electrophoresis on a polyacrylamide gel and transferred to a PVDF membrane with an iBlot system (Thermo-Fisher). The membrane was soaked in Blocking buffer (Licor Biosciences, Lincoln, NE) for 1 hr at room temperature, followed by incubation with anti-mouse antibody against HuCoV-OC43 and anti-rabbit GAPDH antibody as a control for 2 hrs, as described above. The membrane was washed four times with PBS containing 0.2% Tween-20 for 10 min each. Secondary anti-mouse and anti-rabbit IR dyes (Licor Bioscience) at a dilution of 1:5000 in PBS with 0.2% Tweeh-20 were added and incubated for 45 min. We carried out four washings as before and scanned the membrane with an Odyssey IR imaging system (Licor Bioscience). The experiment was replicated twice more and averages of relative intensities of OC43 protein and GAPDH bands were plotted.

### Statistics

The experiments in cell culture are presented as average ± standard deviation. Student unpaired two tail t test was used to compare the mean transcript levels under different treatments. Where multiple treatments were compared, we used one way ANOVA followed by Tukey’s multiple comparison test using GraphPad Prism 9 software (San Diego, CA). A p value of less than 0.05 was considered as significant.

## Results

### Antiviral Activity of SOCS1/3 Antagonist pJAK2 Peptide

**Figure 1:**
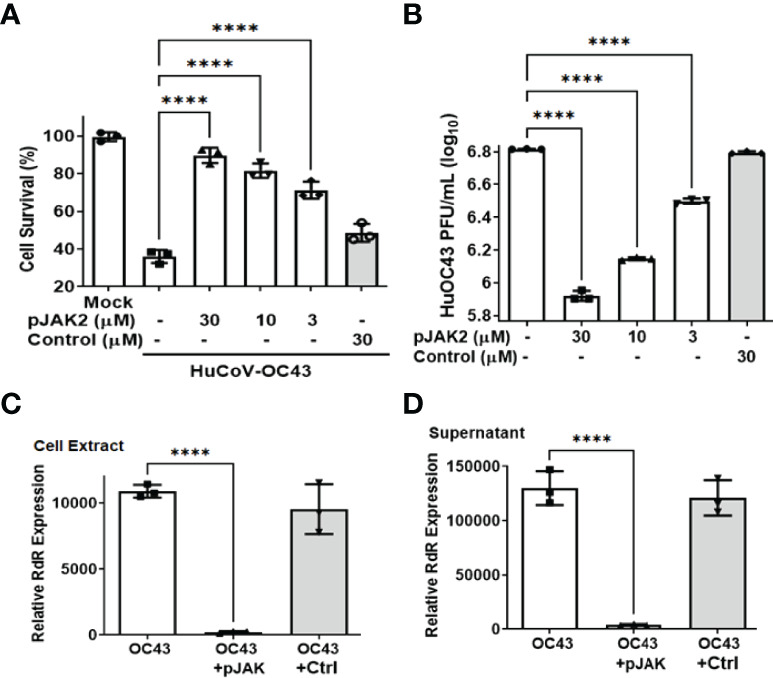
(pJAK2 peptide exhibits antiviral activity against HuCoV-OC43 **(A)**. Protection of cell toxicity caused by HuCoV-OC43. RD cells (human rhabdomyosarcoma cells) were pre-treated with the indicated concentrations of pJAK2 or its control peptide for one hour followed by infection with HuCoV-OC43 at MOI of 0.1 for 1 hour. Cells were washed with PBS and incubated in low serum media for 72 hours. Cell viability was assessed using the CellTiter kit (Promega). p< 0.0001 between different concentrations of pJAK2 peptide and virus only. **(B)**. Antiviral activity of pJAK2 against HuCoV-OC43. RD cells were treated with pJAK2 or control peptide and infected as in **(A)**. At the end of 48 hrs, supernatants were harvested and used in triplicates for plaque assay using Vero E6 cells for 72 hrs. Cells were stained with crystal violet and plaques were counted. The results represent the average of 3 independent experiments. p<0.0001 between different concentrations of pJAK2 peptide and virus only. **(C, D)**. Inhibition of propagation as well as release of HuCoV-OC43 by pJAK2 peptide. RD cells were pre-treated with pJAK2 or its inactive control peptide (both at 30 µM) for one hour followed by infection with OC43 as in **(A)** for 48 hrs. RNA was extracted from cell extract **(C)** and supernatant **(D)**, converted to cDNA and used for qPCR with primers from RNA dependent RNA polymerase (RdR) encoded by OC43. Human β-actin primers were used to calculate relative expression of RdR. (****p<0.0001).

Although vaccines have recently been available for treatment against the highly transmissible and pathogenic SARS-CoV-2, there has been the concern against their effectiveness against the rapidly evolving newer strains. Since treatments for infected patients will continue to be essential, we have tested the antiviral activity of cell penetrating peptides that we previously demonstrated to have shown broad antiviral activity against a number of DNA and RNA viruses (27, 28). Here, we have tested it for antiviral activity against the beta-coronaviruses human OC43 (HuCoV-OC43) and SARS-CoV-2. Since HuCoV-OC43 can be handled under BSL2 conditions, we studied replication of this virus replication in the human rhabdomyosarcoma cell line RD, for most of the initial studies. RD cells were pre-treated with varying concentrations of pJAK2 or its control peptide for 1 hour, followed by infection with HuCoV-OC43 for 1 hr. The cells were washed and incubated with similar concentrations of the peptides in low serum media for 72 hrs. At the end of incubation, the cells surviving were quantitated using the CellTiter kit (**Figure 1A** **)**. In the presence of 30, 10 and 3 μM of pJAK2 there were 90%, 78% and 68% surviving cells, respectively (p<0.0001 for the three concentrations relative to no treatment). This level of survival in cells was associated with complete protection in mice against lethal doses of vaccinia virus, EMCV (28), and influenza virus infection (27). The control peptide showed modest protection as compared with the mock infected cells (p<0.05). Antiviral activity of pJAK2 peptide was verified by assaying the plaque forming units (PFU) in the culture supernatants. RD cells were pre-treated with increasing concentrations of pJAK2 peptide or the control peptide for 1 hr followed by infection with HuCoV-OC43 for 1 hr. We then washed the cells and suspended them in low serum media containing the same amount of peptides for 48 hrs. The supernatants were harvested, serially diluted 10 fold and aliquots were loaded on Vero-E6 cells in triplicate and incubated for 72 hrs. We stained the cells with crystal violet, and the plaques were counted (**Figure 1B**). We noted 8-fold, 4.5-fold and 2-fold decreases in the number of plaques in the presence of pJAK2 peptide at 30, 10 and 3 μM, respectively (p <0.0001 for all versus mock infection). The control peptide led to only a 5% decrease in the plaques formed (p<0.05), suggesting the specificity of pJAK2 action. After deducting the background absorbance in the untreated cells, and extrapolating from the concentrations evaluated, the IC50 was approximately 3 micromolar.

**Figure 2:**
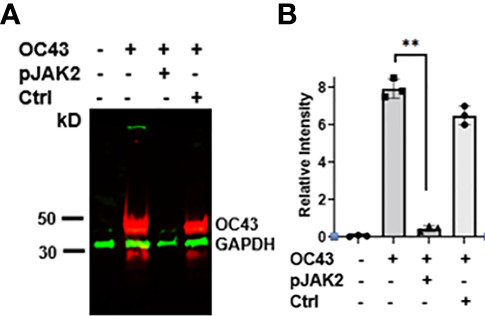
**(A)** Expression of HuCoV-OC43 induced protein is downregulated by pJAK2 peptide. Proteins from cell extracts (equal amounts) of untreated, virus only or the cells infected with the virus in the presence of pJAK2 or control peptide were run on acrylamide gel, transferred to PVDF membrane and probed with primary antibodies to anti-mouse HuCoV-OC43 and anti-rabbit GAPDH (internal control). They were next probed with IR conjugated secondary anti-mouse antibody (red) and anti-rabbit antibody (green). The membrane was scanned in Odyssey IR imaging system (Licor Biosciences). The experiment was repeated using the same conditions two more times. From the three blots, relative intensities between the OC43 and GAPDH bands were calculated using image J and are shown in **(B)** **p < 0.001 between the infected cells and infected cells in the presence of pJAK2 peptide.

**Figure 3:**
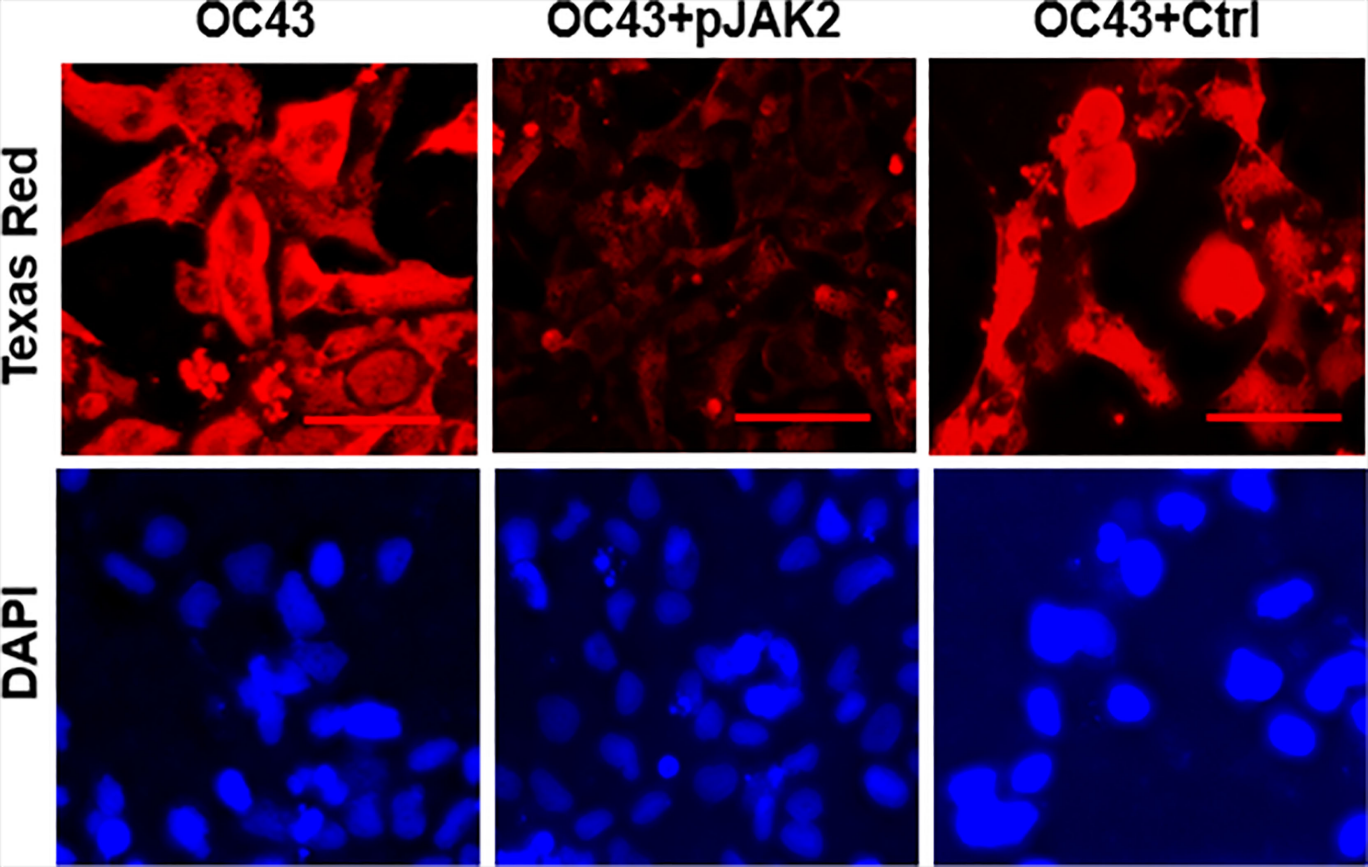
pJAK2 peptide blocks the replication of human coronavirus OC43. RD cells were grown overnight in eight well microscopic slides. They were incubated in low serum medium and treated with pJAK2 or its inactive peptide (both at 30 μM) for 1 hr followed by infection with human CoV OC43 at MOI of 0.1 for 1 hr. The virus was washed, taken in low serum mediun and incubated for 72 hrs. Cells were then fixed, permeabilized and stained with an antibody to the nucleocapsid protein of HuCoV-OC43. Cells were stained with Texas red conjugated secondary anti-mouse antibody and DAPI, followed by fluorescence microscopy. The scale bar represents 50 nm.

To confirm the antiviral effect of pJAK2 peptide, RNA was extracted from cells and supernatants after infection of cells treated with 30μM pJAK2 or the same concentration of control peptide for 48 hrs as described above. RNA was used to synthesize cDNA, which was used for qPCR using the primers from RNA dependent RNA polymerase (RdR) encoded by HuCoV-OC43. Sequence of the primers is shown in **Table 1**. In the presence of pJAK2 peptide, there was a 46-fold decrease in RdR in cell extract, while in the supernatant, there was a 33-fold decrease in RdR RNA (p <0.001 for both) (**Figures 1C, D**). The control peptide led to modest decrease in the levels of RdR RNA that did not reach statistical significance (p>0.05). This suggests that treatment with pJAK2 inhibited both the replication of HuCoV-OC43 in the cells and its release in the supernatant, which is consistent with reduction in PFU noted above. To validate the results from RT-qPCR, we carried out a Western blot using a monoclonal antibody against OC-43 nucleoprotein and a primary antibody to GAPDH. We probed the blot with anti-mouse antibody (red) and anti-rabbit (green) secondary antibodies (**Figure 2A**). We analyzed two more biological replicates by Western blotting and report the averages of ratio of nucleoprotein to GAPDH from these experiments in **Figure 2B**. We noted robust expression of OC43 antigen in cells infected with HuCoV-OC43, which was reduced 19-fold in the simultaneous presence of 30 μM of pJAK2 peptide. The control peptide did not have an appreciable effect on the level of OC-43 protein, suggesting the specific action of pJAK2 peptide. We also validated the propagation of CoV-OC43 by immunohistochemistry. RD cells were grown on eight-well slides overnight, and probed with an antibody to nucleoprotein of HuCoV-OC43, followed by staining with secondary Texas red conjugated anti-mouse antibody and DAPI (**Figure 3**). Examination with fluorescence microscopy revealed accumulation of the HuCoV-OC43 protein around the cells. This accumulation was prevented upon treatment with pJAK2 peptide, but not by the control peptide, suggesting an inhibition of viral replication.

### Induction of SOCS1 and 3 Following Infection With HuCoV-OC43

**Figure 4:**
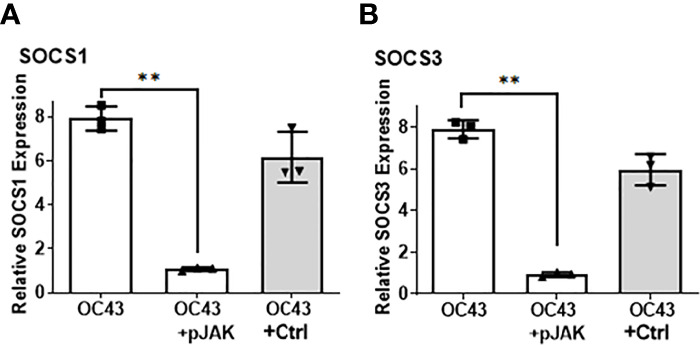
Infection with HuCoV-OC43 induces SOCS1 and SOCS3, which is blocked in the presence of pJAK2 peptide. RD cells were pre-treated with pJAK2 or its inactive control peptide (both at 30 μM) for one hour followed by infection with HuCoV-OC43 at MOI of 0.1 for one hour. The virus was washed and taken in low serum medium and the same concentration of peptides were added to the cells and incubated for 24 hrs. RNA was extracted and used for qPCR with primers for SOCS1 **(A)** and SOCS3 **(B)**. Human β-actin primers were used to calculate relative expression of SOCS1 and SOCS3. (**p <0.001).

Given the protective effect of pJAK2 peptide against HuCoV-OC43 infection, we tested if that effect was caused by blunting of the SOCS1 and/or SOCS3 response following the infection. RD cells were pretreated with pJAK2 or the control peptide followed by infection with HuCoV-OC43, as before. One day later, we isolated RNA from cell extracts for RT-qPCR using the primers specific for human SOCS1 or SOCS3. Infection with HuCoV-OC43 caused an eight-fold induction of SOCS1 (**Figure 4A**) as well as SOCS3 (**Figure 4B**) (p < 0.01 for both), which was prevented in the presence of 30 μM pJAK2. The presence of the control peptide did not affect the expression of SOCS1 or SOCS3. This result suggests that the virus caused an induction of SOCS1 and SOCS3 for its early spread. The presence of pJAK2 that suppresses the activities of SOCS1 and SOCS3 may have resulted in further decrease of replication of HuCoV-OC43.

### Antiviral Action of IFNα-C Peptide and Its Additive Effect With pJAK2 Peptide

**Figure 5:**
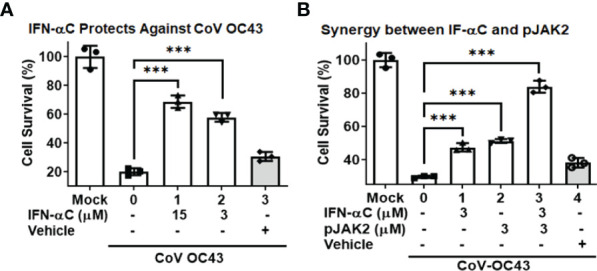
IFN-αC peptide protects against cell toxicity caused by human coronavirus OC43. **(A)** RD cells were pre-treated with the indicated concentrations of IFNα−C or vehicle for one hour followed by infection with HuCoV-OC43 at MOI of 0.1 for 1 hour. Cells were washed with PBS and incubated in low serum media containing the same amount of peptide for 72 hours. Cell viability was assessed using the CellTiter kit (Promega). **(B)** RD cells were pre-treated with IFN-αC, pJAK2 or a combination of the two at 3 μM for one hour and infected with CoV-OC43 at MOI of 0.1 for one hour. The cells were then treated as in **(A)**. ***p<0.0001 between different concentrations of pJAK2 peptide and virus only.

We have previously reported the antiviral action of IFNα−C peptide (which consists of the human IFNα1 C-terminal residues 152-189 conjugated to a lipophilic moiety for cell penetration) against several DNA and RNA viruses, without the associated toxicity seen in parent IFN (25, 38). Therefore, we tested if IFN-αC peptide would prevent cell death caused by HuCoV-OC43. We pre-treated RD cells with different concentrations of IFNα-C or the vehicle (DMSO, used for dissolving the peptide), infected with HuCoV-OC43, washed them and then incubated for 72 hr. At the end of this period, we measured cell survival using CellTiter kit (Promega). There was a 70% ( ± 4%) and 58% ( ± 3%) cell survival noted in the presence of 15 and 3 μM IFN-αC (p < 0.001), while the survival in vehicle treated cells was only slightly elevated relative to infected cells without treatment (p<0.05) thus indicating the antiviral action of IFNα-C (**Figure 5A**). We then tested if the combination of IFN-αC and pJAK2 led to increased survival relative to treatment with single peptides. RD cells treated with 3 μM each of IFN-αC or pJAK2 showed a 50% increase in cell survival relative to infected cells without peptide treatment, while the combined presence of IFN-αC and pJAK2 at the same concentrations resulted in 84% cell survival of infected cells, indicating a greater antiviral protection in the combined presence of the two peptides. The control did not show any protection (**Figure 5B**).

**Figure 6:**
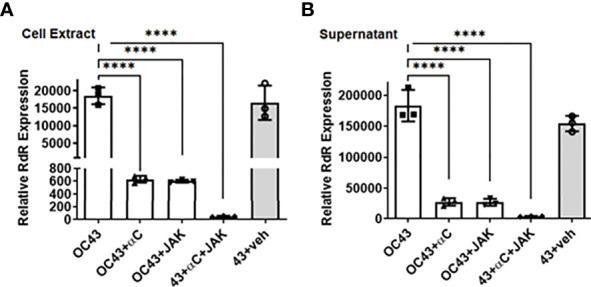
IFNα−C peptide synergizes with pJAK2 peptide in propagation and release of human HuCoV-OC43. RD cells were pre-treated with IFNα−C, pJAK2 peptide, or a combination of the two at 3 μM for 1 hour followed by infection with OC43 at MOI of 0.1 for one hour. The virus was washed and taken in low serum medium with the same concentration of peptides and incubated for 48 hrs. RNA was extracted from cell extract **(A)** and supernatant **(B)**, converted to cDNA and used for qPCR with primers from RNA dependent RNA polymerase (RdR) encoded by OC43. Human β-actin primers were used to calculate relative expression of RdR. (veh, vehicle; ****p<0.0001).

Next, we measured the plaque formation to evaluate the antiviral action of IFN-αC. RD cells were pretreated with increasing concentrations of IFN-αC or the control and infected as described above. At the end of 48 hrs, we harvested the supernatants and measured plaque formation on Vero-E6 cells in serial dilutions. In the presence of 30 μM, 10 μM and 3 μM of IFN-αC, there was a 7.5-fold, 4-fold and 2.3 -fold reduction, respectively, in the number of plaques formed, as compared with untreated cells, while the control peptide showed only a modest reduction in the number of plaques (**Supplementary Figure 1**). We tested the effect of combined IFN-αC and pJAK2 peptides by measuring the viral protein expressed in these cells (**Figure 6**). RD cells were treated with 3 μM each of IFNα−C and pJAK2 separately and also the combination of the two peptides and infected with HuCoV-OC43 as described above. At the end of 48 hr, we harvested the cell extracts and supernatants, extracted RNA, and analyzed the cDNA by qPCR using the primers from the virus encoded RNA dependent RNA polymerase (RdR). IFN-αC and pJAK2 caused a 29.5 ± 2.5 and 31 ± 1.1 fold decrease, respectively, while the combination of the two resulted in 371 ± 65 fold decrease of RdR expression in the cell extracts (p < 0.0001 for the three treatments versus the mock infection). In the supernatants, there was a 6.9 ± 1.7 fold and 7 ± 1.4 fold decrease, in the presence of IFN-αC and pJAK2 peptides, respectively, while the combined treatment resulted in 46.4 ± 7 fold decrease in the expression of the HuCoV-OC43 encoded RNA dependent RNA polymerase. Thus, the combined treatment of IFNα-C and pJAK2 peptides resulted in a synergistic effect in the suppression of both the replication of the virus in the cells and its release in the supernatant. The inhibition observed by RT-qPCR was next verified by Western blots (**Supplemental Figure 2A**). We analyzed cell extracts from cells treated with IFN-αC or the vehicle by Western blot, using antibodies to the viral nucleoprotein and GAPDH as an internal control. The blots were probed with secondary anti-mouse antibodies to detect nucleoprotein (red) and GAPDH (green, internal control). The results of three biological replicates of the relative intensities of OC-43 protein and GAPDH are plotted in **Supplemental Figure 2B**. Treatment with IFNα-C resulted in 17-fold inhibition of the viral protein expression, while the control did not affect the level of the protein, consistent with the other effects of IFN-αC on the replication of HuCoV-OC43. We then investigated inhibition of HuCoV-OC43 by immunohistochemistry. Cells were probed with an antibody to nucleoprotein of HuCoV-OC43, followed by staining with AlexaFluor488 conjugated anti-mouse antibody and DAPI (**Supplemental Figure 3**). Fluorescence microscopy showed that the virus spread around the cells was absent after treatment with IFN-αC peptide, while the vehicle did not affect the spread of the virus. All of these observations confirm the anti-viral activity of both the pJAK2 and IFN-αC peptides and that the combination of the two peptides provides greater protection against HuCoV-OC43.

### Inhibition of SARS-CoV-2 Replication by pJAK2 Peptide

**Figure 7:**
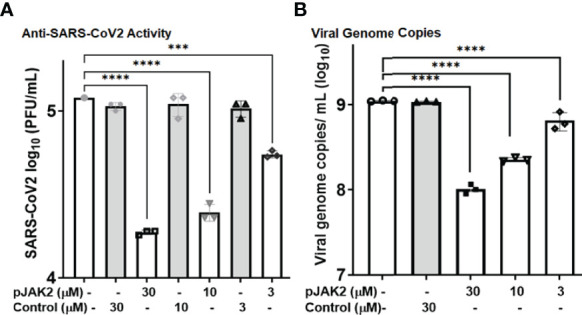
Antiviral activity of pJAK2 against SARS-CoV-2. **(A)** Calu-3 cells were pretreated with pJAK2 or control peptide at 30 µM, 10 µM, and 3 µM for 2hrs prior to infection. Cells were infected in the presence of the peptides at the indicated concentrations with an MOI of 0.01 with SARS-CoV-2 strain UF 1. Following 1hr of infection, inoculum was removed, and cells were rinsed with PBS. Medium was replaced with peptides at indicted concentrations above. Following 24hrs of infection, supernatant was removed and used to perform plaque assays on Vero-E6 cells. After 72hrs postinfection plaque assays were stained and enumerated. **(B)** From the same experiment as in **(A)**, 100 uL of supernatant was removed and used to perform RT-qPCR to detect SARS-CoV-2 RdRp/Hel. ***p < 0.0001; ****p < 0.00001.

While the antiviral effect of the pJAK2 peptide is of medical interest, developing a general treatment for the pandemic SARS-CoV-2 virus is of utmost importance. SARS-CoV-2 strain UF1, a close relative of the original Wuhan strain, was studied under BSL-3 conditions. Human lung adenocarcinoma cells, Calu-3, served as the host cells for replication of this virus. Pre-treatment with increasing concentrations of the pJAK2 peptide was followed by infection with SARS-CoV-2 at an MOI of 0.001 for one hour. We then washed the cells with PBS followed by incubation in medium containing the same amount of peptide. At the end of 24 hr, we removed cells by centrifugation and the supernatants were analyzed for the virus titers. Serial dilutions of these supernatants were seeded in triplicate on Vero-E6 cells and incubated for 72 hr (**Figure 7A**). We stained the dishes with crystal violet and counted the number of plaques. There was a 6.4-fold (p< 0.0001), 4.9-fold (p < 0.0001) and 2.2-fold (p <0.001) reduction in the number of plaques in the presence of 30 μM, 10 μM and 3 μM pJAK2 peptide, respectively. The difference in plaque forming units between infected cells without peptide treatment and control treated cells was not statistically significant. We validated the inhibition of replication using the supernatants from the same treatment from the plaque assay. We extracted RNA for qPCR using primers from RNA dependent RNA polymerase/Helicase encoded by the SARS-CoV-2 (**Table 1**). There was 10.9-fold, 4.9- fold and 1.7-fold reduction in the viral genome detected in supernatants from cells treated with 30 μM, 10 μM; and 3 μM pJAK2 peptide, respectively (p< 0.0001 for all treatments) (**Figure 7B**). The control peptide did not result in a significant decrease of the viral genome indicating the specificity of action of pJAK2 peptide. These results suggests that the pJAK2 peptide can act as an effective antiviral drug for the treatment of SARS-CoV-2 infection.

## Discussion

In this report, we describe the pJAK2 peptide, a SOCS1/3 antagonist peptide with poly-arginine (R9) attached to the N-terminus for cell penetration to make it water soluble and enable easy handling. We have previously used a pJAK2 peptide that carried a palmitoyl-lysine on the N-terminus to make it cell permeable, which was denoted as Lipo-pJAK2. Lipo-pJAK2 peptide administered intraperitoneally in mice for 2 hrs was shown to enter peritoneal cells and various organs of mice (27), thus demonstrating its cell penetration properties. pJAK2 inhibits a number of DNA and RNA viruses, including HSV-1 (24), encephalomyocarditis virus (EMCV), and vaccinia virus (28). The pJAK2 peptide also protected mice against a lethal dose Influenza A PR8 virus, reducing morbidity and mortality and boosting cellular and humoral immune responses (27). Like SARS-CoV-2, influenza A is pandemic respiratory virus. In experiment with Influenza A, we observed adjuvant activity in the pJAK2 peptide against M2e antigen of influenza virus and also against a weak antigen, bovine serum albumin (27). Wang and co-workers reported that pJAK2 enhances antigen presentation in dendritic cells and thus increases the T cell response to protect against gastric cancer (39). As an alternative to the pJAK2 peptide, use of siRNA against SOCS1 in dendritic cells caused increased antigen presentation and increased antiviral activity (40).

A less recognized but an important function of interferons is that most cells express low levels of intracellular IFNβ (41), and a benefit of pJAK2 action was that it increases this basal level of IFNβ, thus augmenting the antiviral response (28). Treatment of L929 cells with 12 μM pJAK2 for 30 min increased the endogenous IFNβ level by 2.5 fold. A similar treatment with pJAK2 did not show any change in the IFNα levels (28). Aside from SOCS, regulatory T cells (Tregs) also play an important role in regulation of immune response. We have shown that SOCS1 is required for a functional peripheral Treg system (20). This has implications in the SOCS antagonist functioning to lower the action of Tregs as well. Taken together, pJAK2 peptide should possess greater immune enhancement by suppressing both the SOCS and Tregs system.

We have shown a direct induction of SOCS1 and SOCS3 by HuCoV-OC43, which is in agreement with previous reports of SOCS induction by beta-coronaviruses spike protein (42). ORF3a protein encoded by SARS-CoV-2 was reported to inhibit IFN signaling by inducing SOCS1 (43). The presence of SOCS1 and/or SOCS3 reduces the induction of various types of IFNs (30, 31, 44). The consequent delayed IFN response results in early viral spread leading to pulmonary and systemic inflammation in critical cases. The presence of pJAK2 peptide results in activation of JAK/STAT signaling leading to activation of the IFN system promoting viral clearance and stimulation of adaptive immune system against the virus (28). Similar to the enhanced SOCS1 and SOCS3 we noted above, other viruses also utilize this pathway to dampen the host immune response (45). Influenza A virus induces SOCS1 and SOCS3 (46, 47), and we have shown strong antiviral properties of pJAK2 against this virus in cell culture as well as in mouse model (27). A number of flavirviruses including Zika virus, West Nile virus, tick-borne encephalitis virus, Japanese encephalitis virus, Dengue virus and Ebola virus, induce SOCS1/3 to dampen the host immune response (reviewed in (48). Human immunodeficiency virus 1 (49), hepatitis B virus (50), hepatitis C virus (51), and the varicella zoster virus (52) have been reported also to use SOCS1 and 3 as virulence factors.

IFNα-C is derived from C-terminus of human IFNα1 residues 152-189. Attachment of a palmitoyl-lysine to the N-terminus of this peptide makes it cell permeable. IFN-αC is species-nonspecific, since the N-terminus of IFNα1 interacts with the IFN receptor for internalization (25, 38). IFNα-C was shown to act through JAK/STAT activation pathway and it lacked the toxicity associated with the parent IFN, as shown by protection against weight loss and lymphocyte suppression (25). The synergistic effect IFN-αC and pJAK2 peptide was noted earlier in protecting C57BL/6 mice against B16F10 cell induced melanoma (25). This result is consistent with the additive effects we noted in protection of these peptides against HuCoV-OC43 reported in **Figures 4**–**6**. Unfortunately, given the expense of operating under BSL3 conditions, we did not have the resources to test the additive effects of IFNα−C and pJAK2 on SARS-Co-V-2, but this experiment is certainly worthwhile in the future.

The parent IFN binds to the extracellular receptor binding domain and is endocytosed. Unlike the parent IFN, the IFN mimetic enters the cell because of the attached cell penetrating domain (poly-Arginine). In our previous studies [reviewed in (25, 33)], we have shown that the internalized IFN mimetic binds to the intracellular domain of IFN receptor in the same region where the parent IFN binds and acts through JAK/STAT pathway similar to the parent IFN. Since the parent IFN binds to the extracellular region of the receptor and the mimetic does not, it makes it difficult to directly compare the specific activities of the parent IFN and the IFN mimetic. Thus, although they may have different specific antiviral activities, the mimetic at higher concentration, can induce an antiviral effect similar to that of the parent IFN, absent the toxicity and species specificity. Details of non-canonical IFN signaling, which are the basis for mimetic development are presented elsewhere (25, 33).

The interferon response presents a double-edged sword with respect to SARS-CoV-2 infections. In early stages, a robust interferon response can limit replication and spread of the virus, whereas, once the virus has reached deep in the lungs, a fulminant inflammatory response is usually associated with poor clinical outcome. The pJAK2 and IFN-αC peptides that we have tested could therefore be useful for early stage injections, but could exacerbate late stage disease. However, the pJAK2 and IFN-αC peptides we propose are likely to be active against rapidly changing variants of SARS-Co-V-2 and other coronaviruses and also against other viruses that have exploited the SOCS1/3 virulence factors.

Cell penetrating peptides are gaining traction as therapeutics (53). The pJAK2 peptide we have tested may be useful as prophylactic as well as a therapeutic. A good choice to deliver the peptides as a prophylactic is to the close contacts of newly infected individuals. We have noted beneficial effects of a SOCS1-KIR peptide in a mouse model of experimental autoimmune uveitis (EAU), where treatment 2 days before immunization as well as treatment one week after immunization by topical treatment protected mice from the disease symptoms (36). Another useful feature of these peptides is their oral bioavailability. For example, oral delivery of the IFNγ peptide protected mice against vaccinia virus infection (34). With a combination of these properties, the peptides we described in this paper may have wide application for viral infection.

## Data Availability Statement

The raw data supporting the conclusions of this article will be made available by the authors, without undue reservation.

## Author Contributions

CA and TG carried out the experiments. CA and AL wrote the manuscript. AL, HJ, and DB supervised the work. All authors contributed to the article and approved the submitted version.

## Funding

This research was supported by Shaler Richardson Professorship endowment, by an unrestricted grant from Research to Prevent Blindness and in part by NIH SIFAR Grant 1S10OD028746 (ASL). Supported in part by an NIH grant AI056152 (HJ). TG was supported by NIH grant T32-AI007110.

## Conflict of Interest

The authors declare that the research was conducted in the absence of any commercial or financial relationships that could be construed as a potential conflict of interest.

## Publisher’s Note

All claims expressed in this article are solely those of the authors and do not necessarily represent those of their affiliated organizations, or those of the publisher, the editors and the reviewers. Any product that may be evaluated in this article, or claim that may be made by its manufacturer, is not guaranteed or endorsed by the publisher.

## Supplementary Material

Supplementary Figure 1 IFNα-C peptide exerts antiviral activity against HuCoV-OC43. RD cells were pre-treated with IFNα-C peptide at the indicated concentrations or the vehicle control (DMSO) for 1 hr followed by infection with HuCoV-OC43 at MOI of 0.1 for 1 hr. Cells were washed and incubated in low serum medium in the presence of same concentrations of peptide or vehicle for 48 hr. The supernatants were harvested and used for plaque assay using Vero-E6 cells, as described in Materials and Methods. p< 0.0001 between different concentrations IFNα-C peptide and virus only.

Supplementary Figure 2 Expression of virus induced protein is downregulated in the presence of IFNα−C peptide. Proteins from cell extracts of untreated, virus only or the cells infected with the virus and treated with IFNα−C peptide or vehicle were separated by electrophoresis and Western blotting was carried out as described for . Using the same conditions, the experiment was repeated two more times. ImageJ was used to measure relative intensities between the OC43 and GAPDH bands, averaged and is shown in **(B)**. **p < 0.001.

Supplementary Figure 3 IFN-αC peptide inhibits the replication of HuCoV-OC43. RD cells were grown overnight in eight well microscopic slides. They were incubated in low serum medium and treated with IFNα−C (30 μM) or vehicle for 1 hr followed by infection with HuCoV-OC43 at MOI of 0.1 for 1 hr. The cells were washed and taken in low serum medium and the same amount of peptides were added back to the cells and incubated for 48 hrs. Cells were then fixed, permeabilized and stained with an antibody to the nucleoprotein of OC43. Cells were stained with AF488 conjugated secondary anti-mouse antibody and DAPI, followed by fluorescence microscopy. Scale bar represents 50 nm.
