# Supplementary figures and images for: Individual and Synergistic Anti-Coronavirus Activities of SOCS1/3 Antagonist and Interferon α1 Peptides
Source: Front Immunol. 2022 Jun 21;13:902956. doi: 10.3389/fimmu.2022.902956 (PMC9254576; doi:10.3389/fimmu.2022.902956)

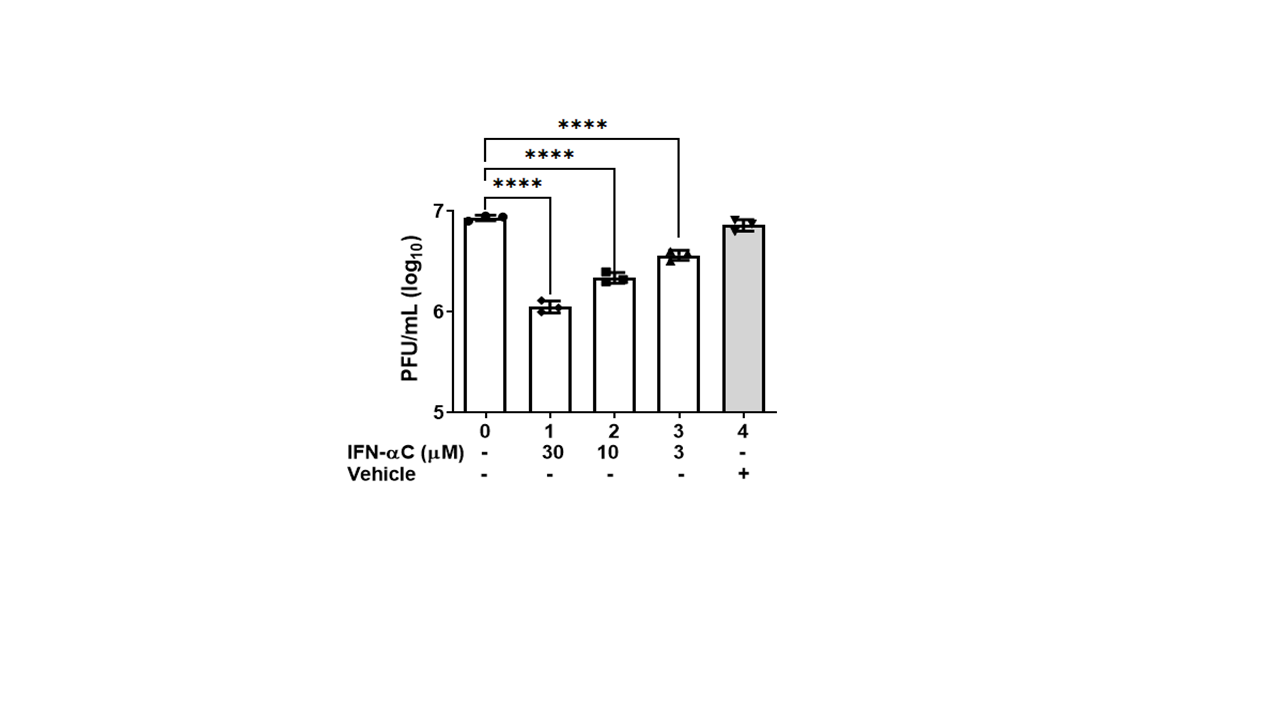

Supplement: Supplementary Figure 1 — IFNα-C peptide exerts antiviral activity against HuCoV-OC43. RD cells were pre-treated with IFNα-C peptide at the indicated concentrations or the vehicle control (DMSO) for 1 hr followed by infection with HuCoV-OC43 at MOI of 0.1 for 1 hr. Cells were washed and incubated in low serum medium in the presence of same concentrations of peptide or vehicle for 48 hr. The supernatants were harvested and used for plaque assay using Vero-E6 cells, as described in Materials and Methods. p< 0.0001 between different concentrations IFNα-C peptide and virus only. [file Image_1.tif]

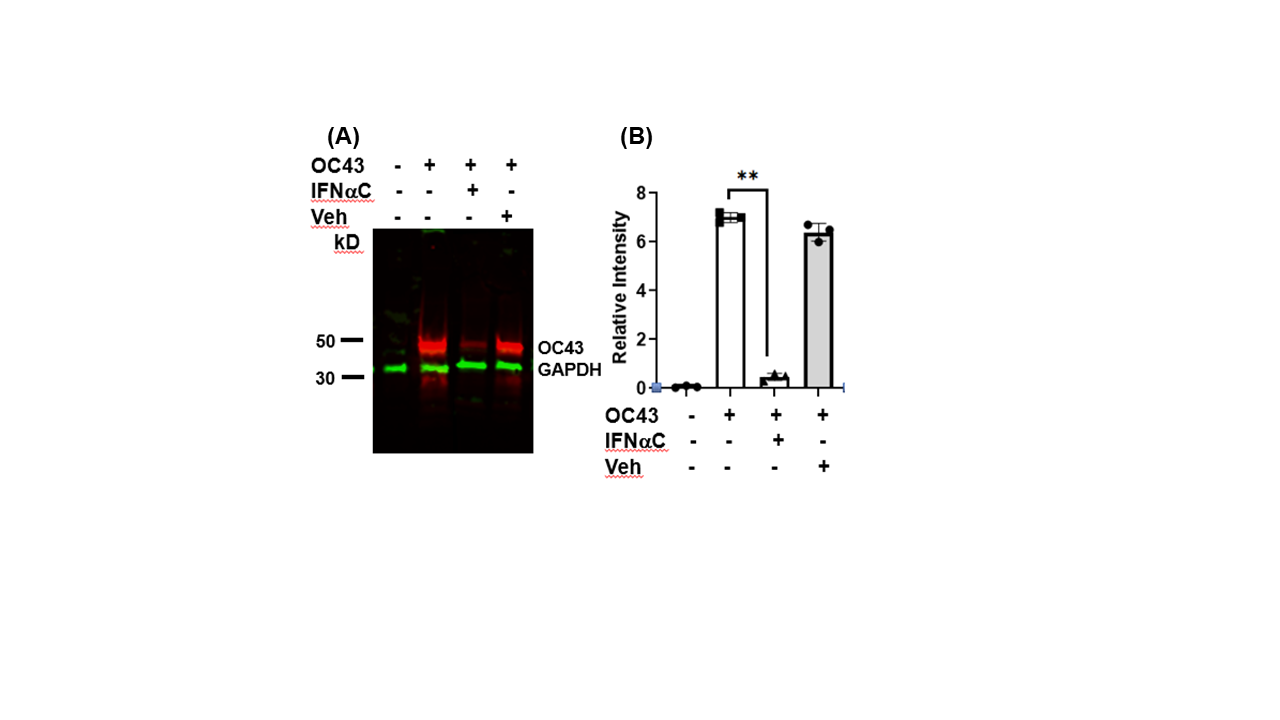

Supplement: Supplementary Figure 2 — Expression of virus induced protein is downregulated in the presence of IFNα−C peptide. Proteins from cell extracts of untreated, virus only or the cells infected with the virus and treated with IFNα−C peptide or vehicle were separated by electrophoresis and Western blotting was carried out as described for . Using the same conditions, the experiment was repeated two more times. ImageJ was used to measure relative intensities between the OC43 and GAPDH bands, averaged and is shown in (B). **p < 0.001. [file Image_2.tif]

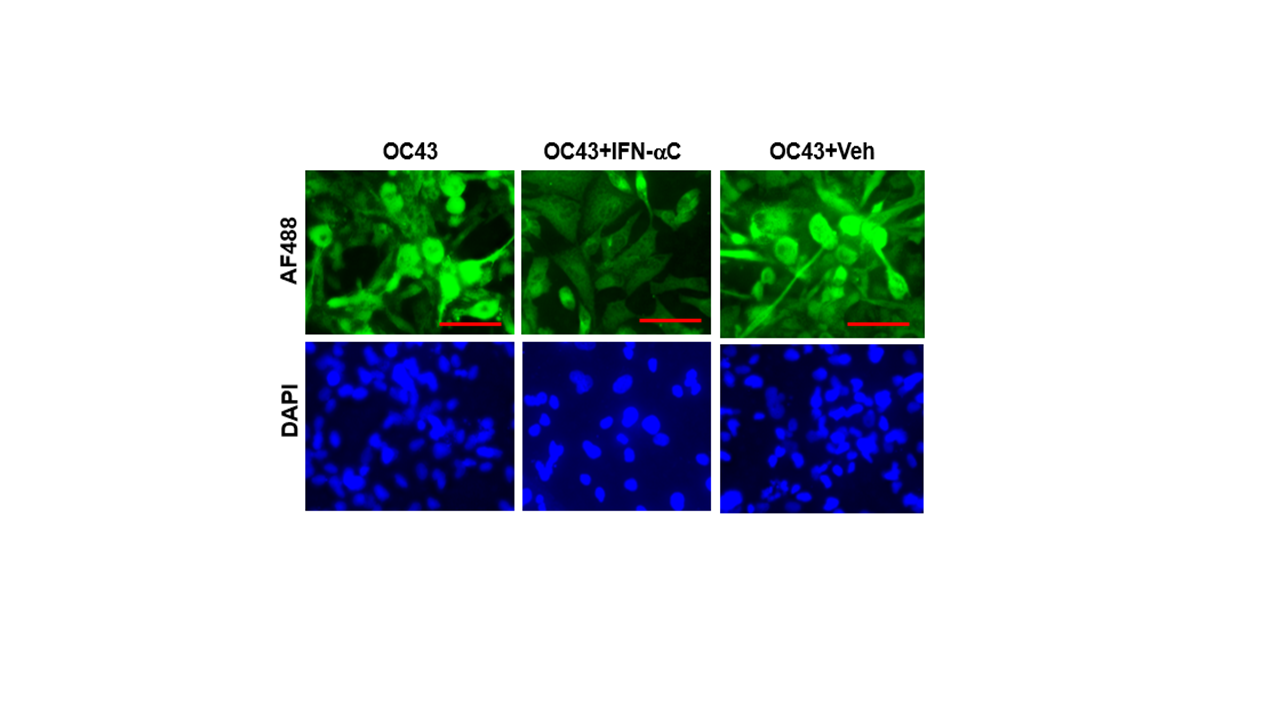

Supplement: Supplementary Figure 3 — IFN-αC peptide inhibits the replication of HuCoV-OC43. RD cells were grown overnight in eight well microscopic slides. They were incubated in low serum medium and treated with IFNα−C (30 μM) or vehicle for 1 hr followed by infection with HuCoV-OC43 at MOI of 0.1 for 1 hr. The cells were washed and taken in low serum medium and the same amount of peptides were added back to the cells and incubated for 48 hrs. Cells were then fixed, permeabilized and stained with an antibody to the nucleoprotein of OC43. Cells were stained with AF488 conjugated secondary anti-mouse antibody and DAPI, followed by fluorescence microscopy. Scale bar represents 50 nm. [file Image_3.tif]
